# Methyl 4-phenyl-1,2,3,3a,4,4a,5,12c-octa­hydronaphtho[1′,2′:3,2]furo[5,4-*b*]pyrrolizine-4a-carboxyl­ate

**DOI:** 10.1107/S1600536810017307

**Published:** 2010-05-15

**Authors:** S. Selvanayagam, B. Sridhar, K. Ravikumar, S. Kathiravan, R. Raghunathan

**Affiliations:** a Department of Physics, Kalasalingam University, Krishnankoil 626 190, India; b Laboratory of X-ray Crystallography, Indian Institute of Chemical Technology, Hyderabad 500 007, India; c Department of Organic Chemistry, University of Madras, Guindy Campus, Chennai 600 025, India

## Abstract

In the title compound, C_26_H_25_NO_3_, both pyrrolidine rings adopt envelope conformations, whereas the dihydro­pyran ring adopts a half-chair conformation. The phenyl ring is oriented at an angle of 27.9 (1)° with respect to the naphthalene ring system. An intra­molecular C—H⋯O hydrogen bond is observed. The crystal packing is stabilized by weak inter­molecular C—H⋯π inter­actions.

## Related literature

For general background to pyrrolizine derivatives, see: Barsoum & Nawar (2003 ▶); Abbas *et al.* (2010 ▶); Anderson & Corey (1977 ▶); Makoni & Sugden (1980 ▶); Laufer *et al.* (1997 ▶). For a related structure, see: Nirmala *et al.* (2009 ▶). For ring-puckering and asymmetry parameters, see: Cremer & Pople (1975 ▶); Nardelli (1983 ▶).
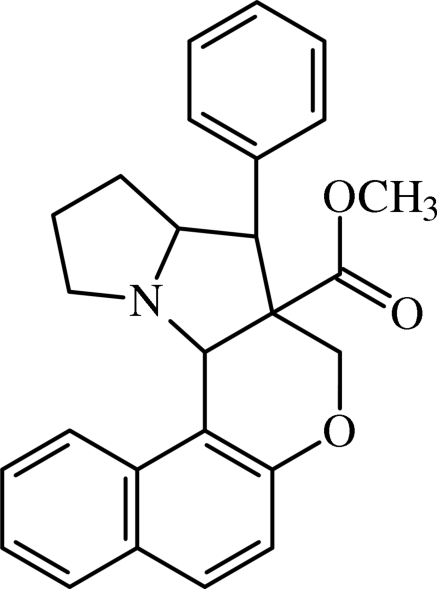


## Experimental

### 

#### Crystal data

C_26_H_25_NO_3_
*M* *_r_* = 399.47
Orthorhombic, 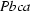

*a* = 15.0117 (12) Å
*b* = 13.3421 (11) Å
*c* = 20.0242 (16) Å
*V* = 4010.6 (6) Å^3^
*Z* = 8
Mo *K*α radiation
μ = 0.09 mm^−1^
*T* = 292 K
0.24 × 0.22 × 0.20 mm

#### Data collection

Bruker SMART APEX CCD area-detector diffractometer
43738 measured reflections
4779 independent reflections
3892 reflections with *I* > 2σ(*I*)
*R*_int_ = 0.030

#### Refinement

*R*[*F* ^2^ > 2σ(*F* ^2^)] = 0.047
*wR*(*F* ^2^) = 0.134
*S* = 1.04
4779 reflections
272 parameters
H-atom parameters constrained
Δρ_max_ = 0.33 e Å^−3^
Δρ_min_ = −0.14 e Å^−3^

### 

Data collection: *SMART* (Bruker, 2001 ▶); cell refinement: *SAINT* (Bruker, 2001 ▶); data reduction: *SAINT*; program(s) used to solve structure: *SHELXS97* (Sheldrick, 2008 ▶); program(s) used to refine structure: *SHELXL97* (Sheldrick, 2008 ▶); molecular graphics: *ORTEP-3* (Farrugia, 1997 ▶) and *PLATON* (Spek, 2009 ▶); software used to prepare material for publication: *SHELXL97* and *PLATON*.

## Supplementary Material

Crystal structure: contains datablocks I, global. DOI: 10.1107/S1600536810017307/ci5092sup1.cif

Structure factors: contains datablocks I. DOI: 10.1107/S1600536810017307/ci5092Isup2.hkl

Additional supplementary materials:  crystallographic information; 3D view; checkCIF report

## Figures and Tables

**Table 1:** Hydrogen-bond geometry (Å, °) *Cg*1 and *Cg*2 are the centroids of the C3–C8 and C19–C24 rings, respectively.

*D*—H⋯*A*	*D*—H	H⋯*A*	*D*⋯*A*	*D*—H⋯*A*
C24—H24⋯O2	0.93	2.59	3.388 (2)	145
C7—H7⋯*Cg*2^i^	0.93	2.82	3.6435 (17)	148
C17—H17*A*⋯*Cg*2^ii^	0.97	2.78	3.7065 (17)	159
C21—H21⋯*Cg*1^iii^	0.93	2.49	3.4174 (18)	175
C26—H26*B*⋯*Cg*1^iv^	0.96	2.58	3.477 (2)	156

Symmetry codes: (i) 
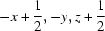
; (ii) 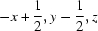; (iii) 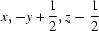; (iv) 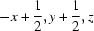.

**Fig. 1.**
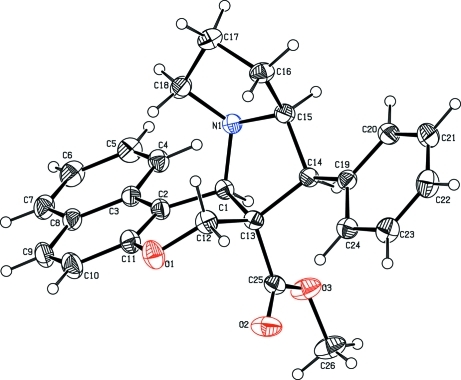
The molecular structure of the title compound, showing the atom-numbering scheme. Displacement ellipsoids are drawn at the 30% probability level

## supplementary crystallographic information

### Comment

Pyrrolizine derivatives posses antimicrobial (Barsoum & Nawar, 2003), anti- inflammatory (Abbas *et al.*, 2010) and antileukemic (Anderson & Corey, 1977) activities. These derivatives are used as inhibitors of blood platelet aggregation (Makoni & Sugden, 1980), dual cyclooxygenase-1 and 5-lipooxygenase (Laufer *et al.*, 1997). In view of these importance, we have undertaken the crystal structure determination of the title compound, a pyrrolizine derivative, and the results are presented here.

The geometry of the pyrrolizine and naphthalene ring system is comparable with that observed in a related structure (Nirmala *et al.*, 2009). The sum of the angles (337.1°) around atom N1 is in accordance with sp^3^ hybridization. There is a short contact (2.19 Å) between atoms H12A and H24, which results in the widening of the C24—C19—C14 angle [124.1 (1)°] from the ideal value of 120°.

The naphthalene ring system (C2–C11) and the phenyl ring (C19–C24) are oriented at an angle of 27.9 (1)°. In the pyrrolizine ring system, both the pyrrolidine rings N1/C1/C13–C15 and N1/C15–C18 adopt envelope conformations; the puckering parameters (Cremer & Pople, 1975) are : q_2_ = 0.396 (1) Å and φ = -107.2 (2)° for N1/C1/C13–C15 ring, and q_2_ = 0.383 (2) Å and φ = -79.1 (2)° for N1/C15–C18 ring. In the N1/C1/C13–C15 ring, atom C13 deviates by 0.620 (1) Å from the least-squares plane through the remaining four atoms, whereas in the ring N1/C15—C18, atom C17 deviates by -0.583 (2) Å from the plane through the remaining four atoms. The dihydropyran ring of the chromene unit adopts a half-chair conformation, with the lowest asymmetry parameter ΔC_2_(C2–C11) of 3.8 (2)° (Nardelli, 1983).

The molecular structure is influenced by an intramolecular C—H···O hydrogen bond. The crystal packing is stabilized by weak intermolecular C—H···π interactions ((Table 1).

### Experimental

A mixture of (*Z*)-methyl-2[(1-formylnaphthalen-2-yloxy)methyl]-3-(4-phenyl) acrylate (20 mmol) and proline (30 mmol) was refluxed in benzene for 20 h and the solvent was removed under reduced pressure. The crude product was subjected to column chromatography to get the pure product. Single crystals were grown by slow evapoartion of a chloroform-methanol (1:1) soution.

### Refinement

H atoms were placed in idealized positions and allowed to ride on their parent atoms, with C—H = 0.93, 0.98, 0.97 and 0.96 Å for aromatic, methine, methylene and methyl H respectively, and Uiso(H) = 1.5U_eq_(C) for methyl H and Uiso(H) = 1.2U_eq_(C) for all other H atoms.

### Crystal data

**Table:**

C_26_H_25_NO_3_	*F*(000) = 1696
*M**_r_* = 399.47	*D*_x_ = 1.323 Mg m^−^^3^
Orthorhombic, *P**b**c**a*	Mo *K*α radiation, λ = 0.71073 Å
Hall symbol: -P 2ac 2ab	Cell parameters from 31182 reflections
*a* = 15.0117 (12) Å	θ = 2.1–27.3°
*b* = 13.3421 (11) Å	µ = 0.09 mm^−^^1^
*c* = 20.0242 (16) Å	*T* = 292 K
*V* = 4010.6 (6) Å^3^	Block, colourless
*Z* = 8	0.24 × 0.22 × 0.20 mm

### Data collection

**Table:**

Bruker SMART APEX CCD area-detector diffractometer	3892 reflections with *I* > 2σ(*I*)
Radiation source: fine-focus sealed tube	*R*_int_ = 0.030
graphite	θ_max_ = 28.0°, θ_min_ = 2.0°
ω scans	*h* = −19→19
43738 measured reflections	*k* = −17→17
4779 independent reflections	*l* = −26→26

### Refinement

**Table:**

Refinement on *F*^2^	Primary atom site location: structure-invariant direct methods
Least-squares matrix: full	Secondary atom site location: difference Fourier map
*R*[*F*^2^ > 2σ(*F*^2^)] = 0.047	Hydrogen site location: inferred from neighbouring sites
*wR*(*F*^2^) = 0.134	H-atom parameters constrained
*S* = 1.04	*w* = 1/[σ^2^(*F*_o_^2^) + (0.0727*P*)^2^ + 0.9686*P*] where *P* = (*F*_o_^2^ + 2*F*_c_^2^)/3
4779 reflections	(Δ/σ)_max_ = 0.001
272 parameters	Δρ_max_ = 0.33 e Å^−^^3^
0 restraints	Δρ_min_ = −0.14 e Å^−^^3^

### Special details

**Table:**

Geometry. All esds (except the esd in the dihedral angle between two l.s. planes) are estimated using the full covariance matrix. The cell esds are taken into account individually in the estimation of esds in distances, angles and torsion angles; correlations between esds in cell parameters are only used when they are defined by crystal symmetry. An approximate (isotropic) treatment of cell esds is used for estimating esds involving l.s. planes.
Refinement. Refinement of *F*^2^ against ALL reflections. The weighted *R*-factor wR and goodness of fit *S* are based on *F*^2^, conventional *R*-factors *R* are based on *F*, with *F* set to zero for negative *F*^2^. The threshold expression of *F*^2^ > σ(*F*^2^) is used only for calculating *R*-factors(gt) etc. and is not relevant to the choice of reflections for refinement. *R*-factors based on *F*^2^ are statistically about twice as large as those based on *F*, and *R*- factors based on ALL data will be even larger.

### Fractional atomic coordinates and isotropic or equivalent isotropic displacement parameters (Å^2^)

**Table:**

	*x*	*y*	*z*	*U*_iso_*/*U*_eq_	
O1	0.10550 (8)	−0.01484 (8)	0.07769 (6)	0.0520 (3)	
O2	0.00482 (7)	0.17668 (10)	0.09040 (6)	0.0580 (3)	
O3	0.10217 (7)	0.27234 (10)	0.14444 (6)	0.0557 (3)	
C1	0.23953 (8)	0.13141 (9)	0.11484 (6)	0.0320 (3)	
H1	0.2427	0.1849	0.1483	0.038*	
C11	0.15871 (10)	−0.02986 (10)	0.13242 (7)	0.0416 (3)	
C10	0.13883 (12)	−0.11691 (11)	0.17021 (9)	0.0519 (4)	
H10	0.0920	−0.1583	0.1574	0.062*	
C9	0.18806 (12)	−0.13999 (11)	0.22501 (8)	0.0498 (4)	
H9	0.1741	−0.1971	0.2495	0.060*	
C8	0.26021 (10)	−0.07888 (10)	0.24562 (7)	0.0403 (3)	
C7	0.31301 (12)	−0.10157 (12)	0.30224 (7)	0.0488 (4)	
H7	0.3009	−0.1592	0.3267	0.059*	
C6	0.38112 (12)	−0.04072 (13)	0.32157 (8)	0.0524 (4)	
H6	0.4144	−0.0562	0.3594	0.063*	
C5	0.40078 (11)	0.04538 (12)	0.28425 (8)	0.0477 (4)	
H5	0.4479	0.0864	0.2971	0.057*	
C4	0.35132 (10)	0.06990 (10)	0.22891 (7)	0.0400 (3)	
H4	0.3655	0.1272	0.2047	0.048*	
C3	0.27917 (9)	0.00946 (9)	0.20814 (6)	0.0346 (3)	
C2	0.22530 (9)	0.03416 (9)	0.15139 (6)	0.0348 (3)	
C13	0.16187 (8)	0.15725 (9)	0.06619 (6)	0.0330 (3)	
C12	0.13359 (10)	0.06083 (11)	0.03088 (7)	0.0415 (3)	
H12A	0.0850	0.0755	0.0004	0.050*	
H12B	0.1831	0.0354	0.0048	0.050*	
N1	0.32208 (7)	0.13550 (8)	0.07478 (5)	0.0347 (3)	
C18	0.36312 (10)	0.04152 (11)	0.05115 (8)	0.0438 (3)	
H18A	0.3189	−0.0111	0.0471	0.053*	
H18B	0.4095	0.0195	0.0815	0.053*	
C17	0.40179 (10)	0.06837 (12)	−0.01673 (8)	0.0450 (3)	
H17A	0.4072	0.0095	−0.0449	0.054*	
H17B	0.4598	0.0996	−0.0122	0.054*	
C16	0.33354 (10)	0.14184 (12)	−0.04488 (7)	0.0431 (3)	
H16A	0.2827	0.1069	−0.0637	0.052*	
H16B	0.3598	0.1844	−0.0789	0.052*	
C15	0.30691 (9)	0.20250 (10)	0.01671 (6)	0.0349 (3)	
H15	0.3468	0.2604	0.0205	0.042*	
C14	0.20917 (8)	0.23942 (10)	0.02349 (6)	0.0327 (3)	
H14	0.2124	0.2983	0.0527	0.039*	
C25	0.08026 (9)	0.20086 (10)	0.10127 (6)	0.0362 (3)	
C26	0.02975 (12)	0.32140 (15)	0.17845 (10)	0.0615 (5)	
H26A	−0.0140	0.3423	0.1464	0.092*	
H26B	0.0519	0.3789	0.2020	0.092*	
H26C	0.0031	0.2757	0.2096	0.092*	
C19	0.16797 (9)	0.27750 (9)	−0.04106 (6)	0.0336 (3)	
C24	0.07883 (9)	0.26452 (10)	−0.05833 (7)	0.0398 (3)	
H24	0.0424	0.2252	−0.0314	0.048*	
C23	0.04346 (11)	0.30931 (11)	−0.11511 (8)	0.0469 (4)	
H23	−0.0165	0.3008	−0.1253	0.056*	
C22	0.09672 (12)	0.36633 (12)	−0.15645 (8)	0.0505 (4)	
H22	0.0733	0.3952	−0.1949	0.061*	
C21	0.18522 (11)	0.38012 (12)	−0.14008 (8)	0.0493 (4)	
H21	0.2215	0.4187	−0.1676	0.059*	
C20	0.22020 (10)	0.33690 (11)	−0.08297 (7)	0.0415 (3)	
H20	0.2797	0.3477	−0.0723	0.050*	

### Atomic displacement parameters (Å^2^)

**Table:**

	*U*^11^	*U*^22^	*U*^33^	*U*^12^	*U*^13^	*U*^23^
O1	0.0578 (7)	0.0420 (5)	0.0561 (6)	−0.0168 (5)	−0.0144 (5)	0.0024 (5)
O2	0.0359 (6)	0.0730 (8)	0.0650 (7)	−0.0104 (5)	0.0103 (5)	−0.0186 (6)
O3	0.0384 (5)	0.0666 (7)	0.0622 (7)	0.0043 (5)	0.0008 (5)	−0.0281 (6)
C1	0.0346 (6)	0.0304 (6)	0.0311 (6)	−0.0025 (5)	−0.0002 (5)	0.0001 (5)
C11	0.0473 (8)	0.0343 (7)	0.0432 (8)	−0.0044 (6)	−0.0007 (6)	−0.0009 (6)
C10	0.0575 (9)	0.0356 (7)	0.0628 (10)	−0.0137 (7)	0.0014 (8)	0.0002 (7)
C9	0.0633 (10)	0.0324 (7)	0.0539 (9)	−0.0039 (6)	0.0109 (8)	0.0079 (6)
C8	0.0518 (8)	0.0320 (6)	0.0369 (7)	0.0056 (6)	0.0111 (6)	0.0022 (5)
C7	0.0648 (10)	0.0430 (8)	0.0387 (7)	0.0134 (7)	0.0112 (7)	0.0103 (6)
C6	0.0618 (10)	0.0594 (10)	0.0359 (7)	0.0139 (8)	−0.0014 (7)	0.0064 (7)
C5	0.0513 (9)	0.0525 (8)	0.0394 (7)	0.0014 (7)	−0.0040 (6)	−0.0010 (6)
C4	0.0466 (7)	0.0383 (7)	0.0351 (7)	−0.0005 (6)	0.0011 (6)	0.0016 (5)
C3	0.0422 (7)	0.0312 (6)	0.0304 (6)	0.0028 (5)	0.0058 (5)	0.0002 (5)
C2	0.0402 (7)	0.0302 (6)	0.0339 (6)	−0.0019 (5)	0.0032 (5)	−0.0004 (5)
C13	0.0328 (6)	0.0348 (6)	0.0315 (6)	−0.0014 (5)	0.0021 (5)	0.0005 (5)
C12	0.0456 (8)	0.0404 (7)	0.0386 (7)	−0.0044 (6)	−0.0037 (6)	−0.0041 (6)
N1	0.0342 (6)	0.0349 (5)	0.0349 (6)	0.0015 (4)	0.0014 (4)	0.0029 (4)
C18	0.0453 (8)	0.0398 (7)	0.0463 (8)	0.0088 (6)	0.0031 (6)	0.0005 (6)
C17	0.0402 (7)	0.0503 (8)	0.0444 (8)	0.0085 (6)	0.0025 (6)	−0.0061 (6)
C16	0.0393 (7)	0.0547 (8)	0.0352 (7)	0.0064 (6)	0.0037 (6)	−0.0012 (6)
C15	0.0321 (6)	0.0379 (7)	0.0347 (6)	−0.0006 (5)	0.0012 (5)	0.0031 (5)
C14	0.0322 (6)	0.0326 (6)	0.0334 (6)	−0.0015 (5)	0.0018 (5)	0.0005 (5)
C25	0.0346 (7)	0.0416 (7)	0.0322 (6)	−0.0029 (5)	0.0038 (5)	0.0030 (5)
C26	0.0476 (9)	0.0748 (12)	0.0619 (10)	0.0107 (8)	0.0039 (8)	−0.0265 (9)
C19	0.0362 (7)	0.0320 (6)	0.0326 (6)	0.0033 (5)	0.0018 (5)	−0.0001 (5)
C24	0.0375 (7)	0.0402 (7)	0.0418 (7)	−0.0004 (5)	0.0008 (6)	0.0021 (6)
C23	0.0458 (8)	0.0465 (8)	0.0483 (8)	0.0014 (6)	−0.0112 (6)	−0.0031 (6)
C22	0.0647 (10)	0.0488 (8)	0.0379 (7)	0.0054 (7)	−0.0101 (7)	0.0055 (6)
C21	0.0567 (9)	0.0477 (8)	0.0436 (8)	0.0007 (7)	0.0060 (7)	0.0121 (6)
C20	0.0390 (7)	0.0426 (7)	0.0429 (7)	−0.0002 (6)	0.0021 (6)	0.0055 (6)

### Geometric parameters (Å, °)

**Table:**

O1—C11	1.3708 (18)	C12—H12B	0.97
O1—C12	1.4408 (18)	N1—C18	1.4751 (18)
O2—C25	1.1974 (17)	N1—C15	1.4842 (16)
O3—C25	1.3285 (17)	C18—C17	1.521 (2)
O3—C26	1.4402 (18)	C18—H18A	0.97
C1—N1	1.4771 (16)	C18—H18B	0.97
C1—C2	1.5049 (17)	C17—C16	1.526 (2)
C1—C13	1.5578 (18)	C17—H17A	0.97
C1—H1	0.98	C17—H17B	0.97
C11—C2	1.3686 (19)	C16—C15	1.5283 (19)
C11—C10	1.418 (2)	C16—H16A	0.97
C10—C9	1.358 (2)	C16—H16B	0.97
C10—H10	0.93	C15—C14	1.5538 (18)
C9—C8	1.417 (2)	C15—H15	0.98
C9—H9	0.93	C14—C19	1.5202 (17)
C8—C7	1.416 (2)	C14—H14	0.98
C8—C3	1.4261 (18)	C26—H26A	0.96
C7—C6	1.362 (3)	C26—H26B	0.96
C7—H7	0.93	C26—H26C	0.96
C6—C5	1.402 (2)	C19—C24	1.3931 (19)
C6—H6	0.93	C19—C20	1.3953 (19)
C5—C4	1.373 (2)	C24—C23	1.390 (2)
C5—H5	0.93	C24—H24	0.93
C4—C3	1.413 (2)	C23—C22	1.380 (2)
C4—H4	0.93	C23—H23	0.93
C3—C2	1.4331 (19)	C22—C21	1.381 (2)
C13—C25	1.5274 (18)	C22—H22	0.93
C13—C12	1.5281 (18)	C21—C20	1.384 (2)
C13—C14	1.5612 (17)	C21—H21	0.93
C12—H12A	0.97	C20—H20	0.93
			
C11—O1—C12	116.86 (11)	C17—C18—H18A	110.9
C25—O3—C26	116.55 (12)	N1—C18—H18B	110.9
N1—C1—C2	114.52 (10)	C17—C18—H18B	110.9
N1—C1—C13	106.26 (10)	H18A—C18—H18B	108.9
C2—C1—C13	112.87 (10)	C18—C17—C16	103.01 (11)
N1—C1—H1	107.6	C18—C17—H17A	111.2
C2—C1—H1	107.6	C16—C17—H17A	111.2
C13—C1—H1	107.6	C18—C17—H17B	111.2
C2—C11—O1	123.80 (13)	C16—C17—H17B	111.2
C2—C11—C10	121.12 (14)	H17A—C17—H17B	109.1
O1—C11—C10	115.07 (13)	C17—C16—C15	102.57 (11)
C9—C10—C11	120.15 (15)	C17—C16—H16A	111.3
C9—C10—H10	119.9	C15—C16—H16A	111.3
C11—C10—H10	119.9	C17—C16—H16B	111.3
C10—C9—C8	121.38 (13)	C15—C16—H16B	111.3
C10—C9—H9	119.3	H16A—C16—H16B	109.2
C8—C9—H9	119.3	N1—C15—C16	105.85 (11)
C7—C8—C9	122.55 (13)	N1—C15—C14	105.50 (10)
C7—C8—C3	119.10 (14)	C16—C15—C14	119.04 (11)
C9—C8—C3	118.34 (13)	N1—C15—H15	108.7
C6—C7—C8	121.36 (14)	C16—C15—H15	108.7
C6—C7—H7	119.3	C14—C15—H15	108.7
C8—C7—H7	119.3	C19—C14—C15	114.57 (10)
C7—C6—C5	119.67 (15)	C19—C14—C13	121.02 (10)
C7—C6—H6	120.2	C15—C14—C13	104.76 (10)
C5—C6—H6	120.2	C19—C14—H14	105.0
C4—C5—C6	120.76 (15)	C15—C14—H14	105.0
C4—C5—H5	119.6	C13—C14—H14	105.0
C6—C5—H5	119.6	O2—C25—O3	123.08 (13)
C5—C4—C3	121.07 (13)	O2—C25—C13	124.91 (13)
C5—C4—H4	119.5	O3—C25—C13	111.97 (11)
C3—C4—H4	119.5	O3—C26—H26A	109.5
C4—C3—C8	118.02 (12)	O3—C26—H26B	109.5
C4—C3—C2	122.33 (12)	H26A—C26—H26B	109.5
C8—C3—C2	119.66 (13)	O3—C26—H26C	109.5
C11—C2—C3	119.25 (12)	H26A—C26—H26C	109.5
C11—C2—C1	120.45 (12)	H26B—C26—H26C	109.5
C3—C2—C1	120.25 (12)	C24—C19—C20	117.45 (12)
C25—C13—C12	108.10 (11)	C24—C19—C14	124.08 (12)
C25—C13—C1	113.39 (10)	C20—C19—C14	118.20 (12)
C12—C13—C1	108.12 (10)	C23—C24—C19	121.11 (13)
C25—C13—C14	110.43 (10)	C23—C24—H24	119.4
C12—C13—C14	117.65 (11)	C19—C24—H24	119.4
C1—C13—C14	99.06 (9)	C22—C23—C24	120.44 (14)
O1—C12—C13	111.73 (11)	C22—C23—H23	119.8
O1—C12—H12A	109.3	C24—C23—H23	119.8
C13—C12—H12A	109.3	C23—C22—C21	119.25 (14)
O1—C12—H12B	109.3	C23—C22—H22	120.4
C13—C12—H12B	109.3	C21—C22—H22	120.4
H12A—C12—H12B	107.9	C22—C21—C20	120.38 (14)
C18—N1—C1	119.55 (11)	C22—C21—H21	119.8
C18—N1—C15	108.94 (10)	C20—C21—H21	119.8
C1—N1—C15	108.61 (10)	C21—C20—C19	121.36 (14)
N1—C18—C17	104.24 (11)	C21—C20—H20	119.3
N1—C18—H18A	110.9	C19—C20—H20	119.3
			
C12—O1—C11—C2	13.1 (2)	C2—C1—N1—C15	149.91 (11)
C12—O1—C11—C10	−168.32 (13)	C13—C1—N1—C15	24.60 (13)
C2—C11—C10—C9	−2.3 (3)	C1—N1—C18—C17	145.27 (12)
O1—C11—C10—C9	179.13 (15)	C15—N1—C18—C17	19.65 (15)
C11—C10—C9—C8	−0.4 (3)	N1—C18—C17—C16	−36.07 (15)
C10—C9—C8—C7	−179.52 (15)	C18—C17—C16—C15	38.40 (15)
C10—C9—C8—C3	1.3 (2)	C18—N1—C15—C16	4.57 (14)
C9—C8—C7—C6	−178.96 (15)	C1—N1—C15—C16	−127.17 (11)
C3—C8—C7—C6	0.2 (2)	C18—N1—C15—C14	131.61 (11)
C8—C7—C6—C5	−1.1 (2)	C1—N1—C15—C14	−0.13 (13)
C7—C6—C5—C4	0.9 (2)	C17—C16—C15—N1	−26.71 (14)
C6—C5—C4—C3	0.2 (2)	C17—C16—C15—C14	−145.10 (12)
C5—C4—C3—C8	−1.2 (2)	N1—C15—C14—C19	−159.08 (10)
C5—C4—C3—C2	178.77 (14)	C16—C15—C14—C19	−40.51 (16)
C7—C8—C3—C4	0.96 (19)	N1—C15—C14—C13	−24.14 (13)
C9—C8—C3—C4	−179.87 (13)	C16—C15—C14—C13	94.43 (13)
C7—C8—C3—C2	−178.97 (12)	C25—C13—C14—C19	−72.42 (14)
C9—C8—C3—C2	0.20 (19)	C12—C13—C14—C19	52.29 (16)
O1—C11—C2—C3	−177.76 (13)	C1—C13—C14—C19	168.34 (11)
C10—C11—C2—C3	3.8 (2)	C25—C13—C14—C15	156.27 (11)
O1—C11—C2—C1	4.7 (2)	C12—C13—C14—C15	−79.02 (14)
C10—C11—C2—C1	−173.79 (13)	C1—C13—C14—C15	37.03 (12)
C4—C3—C2—C11	177.36 (13)	C26—O3—C25—O2	−0.3 (2)
C8—C3—C2—C11	−2.7 (2)	C26—O3—C25—C13	177.64 (14)
C4—C3—C2—C1	−5.09 (19)	C12—C13—C25—O2	−15.40 (19)
C8—C3—C2—C1	174.83 (11)	C1—C13—C25—O2	−135.26 (15)
N1—C1—C2—C11	−110.51 (14)	C14—C13—C25—O2	114.60 (16)
C13—C1—C2—C11	11.26 (18)	C12—C13—C25—O3	166.74 (12)
N1—C1—C2—C3	71.97 (15)	C1—C13—C25—O3	46.88 (15)
C13—C1—C2—C3	−166.26 (11)	C14—C13—C25—O3	−63.26 (14)
N1—C1—C13—C25	−154.82 (10)	C15—C14—C19—C24	144.48 (13)
C2—C1—C13—C25	78.87 (13)	C13—C14—C19—C24	17.48 (19)
N1—C1—C13—C12	85.34 (12)	C15—C14—C19—C20	−41.57 (16)
C2—C1—C13—C12	−40.98 (14)	C13—C14—C19—C20	−168.57 (12)
N1—C1—C13—C14	−37.80 (12)	C20—C19—C24—C23	−0.1 (2)
C2—C1—C13—C14	−164.12 (10)	C14—C19—C24—C23	173.86 (13)
C11—O1—C12—C13	−45.96 (17)	C19—C24—C23—C22	1.2 (2)
C25—C13—C12—O1	−64.37 (14)	C24—C23—C22—C21	−1.3 (2)
C1—C13—C12—O1	58.76 (15)	C23—C22—C21—C20	0.2 (2)
C14—C13—C12—O1	169.77 (11)	C22—C21—C20—C19	0.9 (2)
C2—C1—N1—C18	24.13 (16)	C24—C19—C20—C21	−0.9 (2)
C13—C1—N1—C18	−101.18 (13)	C14—C19—C20—C21	−175.28 (13)

### Hydrogen-bond geometry (Å, °)

**Table:**

Cg1 and Cg2 are the centroids of the C3–C8 and C19–C24 rings, respectively.

**Table:**

*D*—H···*A*	*D*—H	H···*A*	*D*···*A*	*D*—H···*A*
C24—H24···O2	0.93	2.59	3.388 (2)	145
C7—H7···Cg2^i^	0.93	2.82	3.6435 (17)	148
C17—H17A···Cg2^ii^	0.97	2.78	3.7065 (17)	159
C21—H21···Cg1^iii^	0.93	2.49	3.4174 (18)	175
C26—H26B···Cg1^iv^	0.96	2.58	3.477 (2)	156

Symmetry codes: (i) −*x*+1/2, −*y*, *z*+1/2; (ii) −*x*+1/2, *y*−1/2, *z*; (iii) *x*, −*y*+1/2, *z*−1/2; (iv) −*x*+1/2, *y*+1/2, *z*.
